# Genome of *Ca*. Pandoraea novymonadis, an Endosymbiotic Bacterium of the Trypanosomatid *Novymonas esmeraldas*

**DOI:** 10.3389/fmicb.2017.01940

**Published:** 2017-10-04

**Authors:** Alexei Y. Kostygov, Anzhelika Butenko, Anna Nenarokova, Daria Tashyreva, Pavel Flegontov, Julius Lukeš, Vyacheslav Yurchenko

**Affiliations:** ^1^Life Science Research Centre, Faculty of Science, University of Ostrava, Ostrava, Czechia; ^2^Zoological Institute of the Russian Academy of Sciences, St. Petersburg, Russia; ^3^Biology Centre, Institute of Parasitology, Czech Academy of Sciences, České Budějovice, Czechia; ^4^Faculty of Sciences, University of South Bohemia, České Budějovice, Czechia; ^5^Institute for Information Transmission Problems, Russian Academy of Sciences, Moscow, Russia; ^6^Institute of Environmental Technologies, Faculty of Science, University of Ostrava, Ostrava, Czechia

**Keywords:** bacterial endosymbiont, *Pandoraea*, phylogenomics, metabolism, Trypanosomatidae

## Abstract

We have sequenced, annotated, and analyzed the genome of *Ca*. Pandoraea novymonadis, a recently described bacterial endosymbiont of the trypanosomatid *Novymonas esmeraldas.* When compared with genomes of its free-living relatives, it has all the hallmarks of the endosymbionts’ genomes, such as significantly reduced size, extensive gene loss, low GC content, numerous gene rearrangements, and low codon usage bias. In addition, *Ca*. P. novymonadis lacks mobile elements, has a strikingly low number of pseudogenes, and almost all genes are single copied. This suggests that it already passed the intensive period of host adaptation, which still can be observed in the genome of *Polynucleobacter necessarius*, a certainly recent endosymbiont. Phylogenetically, *Ca*. P. novymonadis is more related to *P. necessarius*, an intracytoplasmic bacterium of free-living ciliates, than to *Ca*. Kinetoplastibacterium spp., the only other known endosymbionts of trypanosomatid flagellates. As judged by the extent of the overall genome reduction and the loss of particular metabolic abilities correlating with the increasing dependence of the symbiont on its host, *Ca*. P. novymonadis occupies an intermediate position *P. necessarius* and *Ca*. Kinetoplastibacterium spp. We conclude that the relationships between *Ca*. P. novymonadis and *N. esmeraldas* are well-established, although not as fine-tuned as in the case of Strigomonadinae and their endosymbionts.

## Introduction

*Pandoraea* is a genus of Gram-negative rod-shaped β-proteobacteria belonging to the family Burkholderiaceae of the order Burkholderiales. Members of this genus are phenotypically diverse, reflecting a wide spectrum of life strategies. Several species of these microorganisms were documented as opportunistic pathogens in cystic fibrosis patients or in individuals after lung transplantation (Coenye et al., 2000; Stryjewski et al., 2003). Besides, a number of *Pandoraea* spp. (including some pathogenic ones) were isolated from environmental samples such as soils, hen dung, and oxic water layer above a sulfide-containing sediment (Coenye et al., 2000; Anandham et al., 2010; Sahin et al., 2011). These free-living species participate in the biodegradation of various organic substances (including important pollutants) or perform chemosynthesis by oxidation of heterotrophic sulfur (Okeke et al., 2002; Graff and Stubner, 2003; Ozaki et al., 2007; Liz et al., 2009; Kumar et al., 2015; Jeong et al., 2016).

Previously, we discovered a new species of *Pandoraea*, which, in contrast to its relatives, is an intracellular symbiont of the flagellate *Novymonas esmeraldas* (Kinetoplastea: Trypanosomatidae) (Flegontov et al., 2016). This endosymbiosis appears to have been established relatively recently as judged by the fact that neither of the two participants has close relatives involved in similar relationships. In addition, the division of *Ca.* Pandoraea novymonadis is not synchronized with that of the host cell. As a result, the number of endosymbionts per *Novymonas* cell is unstable and bacteria-free trypanosomatids appear at a relatively high frequency of ∼6%. We hypothesized that the endosymbiosis is favorable for *N. esmeraldas*, since large-scale cloning experiments did not reveal any aposymbiotic clone (Flegontov et al., 2016).

All other studied endosymbioses in trypanosomatids are restricted to flagellates of the subfamily Strigomonadinae (Votýpka et al., 2014) and bacteria *Ca*. Kinetoplastibacterium spp. (Burkholderiales: Alcaligenaceae). These relationships seem to have been established earlier in evolution. As judged from the phylogenies of the prokaryotic and eukaryotic partners, the origin of this endosymbiosis was a single event (Du et al., 1994; Teixeira et al., 2011). The long evolution of *Kinetoplastibacterium* resulted in “one bacterium per host cell” arrangement with fine-tuned mechanisms synchronizing their division (Motta et al., 2010; Catta-Preta et al., 2015). The bacterium provides its host with essential nutrients and is remunerated with a direct access to the ATP-producing glycosomes (Motta et al., 1997; de Souza and Motta, 1999; Alves et al., 2011, 2013a).

The free-living hypotrichous ciliates (*Euplotes aediculatus* and related species) with their intracytoplasmic bacterium *Ca. Polynucleobacter necessarius* (hereafter conventionally called *P. necessarius*) represent yet another endosymbiotic association in a protist, reminiscent of the *Novymonas*/*Pandoraea* system. Although the ciliates are evolutionary extremely distant from trypanosomatids and represent a different eukaryotic supergroup (SAR versus Excavata), their endosymbiont *P. necessarius* belongs to the same β-proteobacterial family *Burkholderiaceae*. This endosymbiosis seems to be quite recent, since there is a very closely related free-living bacterium formally attributed to a separate species *P. asymbioticus*, but showing 99% identity with *P. necessarius* in their 16S rRNA gene sequences (Vannini et al., 2007). Another sign of the relatively nascent nature of these relationships is that *P. necessarius* is apparently a substitute for a more ancient symbiont (*Ca.* Protistobacter heckmanni), another representative of the family *Burkholderiaceae*, which can be found in some *Euplotes* isolates (Vannini et al., 2012, 2013).

Obligate intracellular bacterial symbionts demonstrate similar patterns of genome evolution: reduction of its size, decrease in GC content, elevated evolutionary rate, loss of genes from certain functional groups (transcriptional regulation, DNA repair, etc.), shrinkage of the repertoire of metabolic capabilities, gene transfer to host’s nucleus, and others (Moya et al., 2008; Nowack and Melkonian, 2010; McCutcheon and Moran, 2011). At early phase of endosymbiosis these changes are accompanied by the expansion of mobile genetic elements, pseudogenization, and multiple genomic rearrangements (Ochman and Davalos, 2006; Toh et al., 2006; Burke and Moran, 2011). In the case of Strigomonadinae*/Ca.* Kinetoplastibacterium, all the above-mentioned traits typical of ancient endosymbiotic associations can be observed (Alves et al., 2013b). The comparison of genomes of *P. necessarius* and *P. asymbioticus* revealed only a limited genome size reduction (∼28% on DNA and ∼34% on the protein level) with a substantial pseudogenization (∼18%), but without any mobile elements (Meincke et al., 2012; Boscaro et al., 2013).

While the host of *Ca.* P. novymonadis is closely related to that of *Ca.* Kinetoplastibacterium spp., the bacterium itself is phylogenetically closer to *Polynucleobacter*. In order to understand the nature of endosymbiotic relationships, their underlying mechanisms and routes of adaptation in the *Novymonas*/*Pandoraea* system, we analyzed the genome of *Ca.* P. novymonadis and compared it with both endosymbiotic systems discussed above.

## Materials and Methods

### Establishing Aposymbiotic Strain of *Novymonas esmeraldas*

The strain E262AT.01 of *N. esmeraldas* was cultivated at 27°C in RPMI-1640 medium (Sigma-Aldrich, St. Louis, MO, United States) supplemented with heat-inactivated 10% fetal bovine serum (FBS; Thermo Fisher Scientific, Waltham, MA, United States). At the logarithmic phase of growth, cells from the 10 ml culture aliquots were pelleted by centrifugation at 1,500 ×*g* for 10 min and re-suspended in the fresh RPMI-1640 medium containing 10, 50, 125, 250, or 500 μg/ml of azithromycin (Barry et al., 2004). This macrolide antibiotic was chosen because of its ability to cross eukaryotic plasma membrane, accumulate in the cytoplasm at high concentration, and retain its activity under these conditions (Maurin and Raoult, 2001; Carryn et al., 2003). The presence/absence of bacterial endosymbionts was monitored after 7 and 14 days of incubation by fluorescent *in situ* hybridization with universal bacteria-specific probe Eub338 (5′-GCTGCCTCCCGTAGGAGT-3′) labeled with 5′-Cy3 fluorescent dye, as described previously (Kostygov et al., 2016). After 14 days incubation with 10 and 50 μg/ml of azithromycin, all observed *N. esmeraldas* cells were free of endosymbionts, while at the higher concentrations of the antibiotic trypanosomatid cells died. The bacteria-free cultures were pelleted and transferred to a fresh azithromycin-free medium. The strain obtained with 10 μg/ml of azithromycin (hereafter named E262-AZI) displayed better growth and was used for all the subsequent experiments. The absence of bacteria in the culture was also confirmed by PCR with universal eubacterial 16S rRNA primers P1seq and 1486R, with the original bacteria-containing strain (hereafter named E262-wt) used as a positive control (Teixeira et al., 2011).

Given the significant deceleration of E262-AZI growth as compared to the E262-wt, for the subsequent work we switched from RPMI to a more nutrient-rich medium, M199 (Sigma-Aldrich, St. Louis, MO, United States) supplemented with 10% FBS, 2 μg/ml hemin (Jena Bioscience, Jena, Germany), 2 μg/ml biopterin, 100 units/ml of penicillin, and 100 μg/ml of streptomycin (all from Thermo Fisher Scientific, Waltham, MA, United States). In these conditions, E262-AZI was able to propagate at higher rate, comparable to that of E262-wt.

### Genomic DNA Isolation and Sequencing

Total genomic DNA was isolated from ∼10^9^ cells of the strains E262-wt and E262-AZI of *N. esmeraldas* using the DNeasy Blood and Tissue Kit (Qiagen, Hilden, Germany) according to the manufacturer’s protocol. The genome of the wild-type *N. esmeraldas* was sequenced using a combination of Illumina Technologies: HiSeq 2000 (Macrogen Inc., Seoul, South Korea) and MiSeq (Palacký University, Olomouc, Czechia), yielding 47,024,780 reads with 145× average coverage and 21,715,370 reads with 160× average coverage, respectively. The genome of the aposymbiotic *N. esmeraldas* was sequenced solely with the Illumina MiSeq technology, resulting into 17,834,848 of reads with 136× average coverage. The lengths of the obtained paired-end reads were 100 nt for the HiSeq and 300 nt for the MiSeq sequences.

### Genome Assembly and Annotation

DNA sequencing reads were processed using BBtools package v.36.02^1^. The reads were merged and quality-trimmed using BBmerge with the quality threshold of 20. Non-merged reads were quality-trimmed using BBduk with the same parameters. The quality of raw and trimmed reads was assessed using FASTQC program v.0.11.5^2^.

The genome assembly for both strains was performed using Spades Genome assembler v.3.9.0 with recommended options (Bankevich et al., 2012). Genomic reads of E262-wt were mapped onto the contigs of the aposymbiotic E262-AZI and the remaining reads were used for assembling the endosymbiont genome. However, a read mapping rate was low (∼50%) and the obtained assembly contained both endosymbiont and host contigs. Hence, we decided to use other methods for identification of the bacterial contigs. Firstly, each of E262-wt contigs was used as a query in BLAST searches against the custom database composed of *Pandoraea* spp. and trypanosomatid genomes. The BLASTN program from the BLAST package v.2.2.31+ (Camacho et al., 2009) was used with an *E*-value cut-off of 10^-5^ and other settings left as default. The total length of a BLAST alignment per contig was calculated using custom Ruby script. For every contig, the query coverage with *Pandoraea* hits was divided by that with trypanosomatid hits. The values above 1 were considered as evidencing the bacterial origin. Secondly, we checked the absence of the putative endosymbiont contigs in the E262-AZI assembly using the BLASTN program as above. The best hits for the presumed bacterial contigs were those with low coverage (∼1×), probably representing technical contamination during sequencing of the E262-AZI sample. Thirdly, the read coverage was considered for distinguishing contigs of *N. esmeraldas* and *Ca.* P. novymonadis. Typically, a cell of this trypanosomatid bears several endosymbionts in the cytoplasm (Kostygov et al., 2016), and each of them might have multiple copies of the bacterial genome. Therefore, the read coverage of *Ca.* P. novymonadis contigs is expected to be higher than that of *N. esmeraldas* contigs. Indeed, the mean coverage per position in the putative bacterial contigs was ∼874× while the remaining ones had only ∼25× read coverage. In addition, the contigs of different origin could be discriminated by their GC content. Trypanosomatid contigs had ∼65% GC, while those of the endosymbiont were only 43–49% GC-rich. This is in agreement with the observation that endosymbiotic genomes usually have lower GC content than the genomes of their hosts (Moran et al., 2008). It should be noted that GC-rich sequences are generally harder to sequence than AT-rich, and this effect may impact the coverage difference and result in overestimation of the bacterial load. Lastly, the Bandage software^3^, analyzing assembly using a BLAST-based approach, was used. The program created contig graphs, showing that all the putative endosymbiont contigs may compose a single circular chromosome (under assumption that the two shortest bacterial contigs having double coverage, as compared to longer ones are duplicated). This also evidenced that our assembly was complete. Despite the results of the Bandage analysis, we were unable to assemble the bacterial contigs into one chromosome due to some ambiguities. Genome completeness analysis was performed using BUSCO software (Simão et al., 2015) with bacteria, proteobacteria, and betaproteobacteria universal gene datasets and the predicted *Ca*. P. novymonadis proteins.

Parameters of the genome assemblies were estimated using QUAST v.4.3 (Gurevich et al., 2013). DNA reads were mapped on the assemblies using Bowtie2 v.2.2.9 (Langmead and Salzberg, 2012), with the “–very-fast” option. The structural and functional annotation of the *Ca.* P. novymonadis genome was obtained using Prokka package v.1.12-beta (Seemann, 2014), signal peptides were predicted using SignalP v.4.1 (Petersen et al., 2011).

This Whole Genome Shotgun project has been deposited at DDBJ/ENA/GenBank under the accession MUHY00000000. The version described in this paper is version MUHY01000000. The raw reads are available at the NCBI Sequence Read Archive under the accession no. SRR5280512.

### Gene Family Inference and Analysis

The inference of protein orthologous groups (OGs) was performed with OrthoFinder v.1.1.3 (Emms and Kelly, 2015) using a dataset of 23 bacterial genomes, including *Ca.* P. novymonadis sequenced in this study, 13 other *Pandoraea* spp., 5 *Ca.* Kinetoplastibacterium spp., 2 *Polynucleobacter* spp., *Cupriavidus basilensis*, and *Burkholderia cepacia* available in GenBank (**Supplementary Table S1**). Gene family gains and losses were mapped on the reference species tree using the COUNT software with the Dollo parsimony algorithm (Csuros, 2010) as described elsewhere (Flegontov et al., 2016). Using UpSetR package for R^4^ and a custom Python script we found OGs exclusively shared between *Ca.* P. novymonadis and the following groups of species: (i) *C. basilensis* and *B. cepacia*, (ii) *Polynucleobacter* spp., (iii) *Pandoraea* spp., and (iv) *Ca.* Kinetoplastibacterium spp. Putative annotations for the *Ca.* P. novymonadis-specific proteins were inferred using HHpred v.2.0.16 against Pfam-A database and *E*-value cut-off set to 1 (Soding et al., 2005).

### Phylogenomic Analysis

In the 16 bacterial strains selected for phylogenetic inference (**Supplementary Table S1**) 556 shared OGs contained only one gene. The amino acid sequences of each single gene were aligned using L-INS-i algorithm in MAFFT v. 7.310 (Katoh and Standley, 2013). The resulting alignments were trimmed in Gblocks v.0.91b with relaxed parameters (-b3 = 8, -b4 = 2, -b5 = h) and then used for phylogenetic reconstruction in IQ-TREE v.1.5.3 with LG + I + G4 + F model and 1,000 ultrafast bootstrap replicates (Minh et al., 2013; Nguyen et al., 2015). The amino acid substitution model had been selected in the same program using the supermatrix concatenated from the individual alignments of all 556 genes (Kalyaanamoorthy et al., 2017). To estimate the resolution power of single genes, for each of the reconstructed trees the average bootstrap support was calculated. Setting 70% as a threshold, we selected 119 genes, which constituted the final dataset. The alignments of these genes were concatenated, producing a supermatrix with 54,345 characters. Maximum-likelihood tree was reconstructed using IQ-Tree with LG + I + G4 + F model and 1,000 standard bootstrap replicates. Bayesian inference of phylogeny was performed in MrBayes v. 3.2.6 (Ronquist et al., 2012) under mixed model prior, empirical amino acid frequencies, and heterogeneity of rates across sites assessed using Γ-distribution and proportion of invariant sites. The analysis was run for 100,000 generations with sampling every 10th of them. The chains demonstrated efficient mixing and the two runs converged at the early phase of the analysis (after 2,500 generations). As set by default, 25% samples were discarded as burn-in.

### Metabolic Pathways Analysis

For the comparative metabolic study an automatic assignment of KEGG Orthology (KO) identifiers to the proteins of 19 bacterial strains including *Ca.* P. novymonadis (**Supplementary Table S1**) was completed using BlastKOALA v.2.1 (Kanehisa et al., 2016). The search was performed against a non-redundant pangenomic databases of prokaryotes on the genus level and of eukaryotes on the family level. KEGG Mapper v.2.8 was used for the reconstruction of metabolic pathways and their comparison (Kanehisa, 2017).

The search for lipolytic enzymes was performed using BLASTP with an *E*-value of 10^-20^ with the lipase and esterase sequences from the study of Arpigny and Jaeger as a query and annotated proteins of *Ca.* P. novymonadis and other bacteria as a database (Arpigny and Jaeger, 1999). In the case of *Ca.* P. novymonadis the *E*-value threshold was relaxed to 10^-10^.

### Synteny Analysis

The overall level of synteny in *Ca.* P. novymonadis as compared to other species of interest was studied using the reference dataset of 11 bacteria (**Supplementary Table S1**). Syntenic regions were inferred and visualized using SyMAP v.4.2 (Soderlund et al., 2011). The settings were as follows: minimum number of anchors required to define a synteny block, 7; overlapping (or nearby) synteny blocks were automatically merged into larger blocks, and only the larger block was kept if two synteny blocks overlapped on a chromosome.

### Search for Pseudogenes, Phages, and Mobile Elements

Pseudogenes in *Ca.* P. novymonadis genome were identified using BLASTX with an *E*-value cut-off of 1 against the dataset of annotated proteins of *C. basilensis, B. cepacia*, and *Pandoraea* spp. (**Supplementary Table S1**). Prior to homology searches, *Ca.* P. novymonadis genes were masked with Maskfasta script from BEDTtools package v. 2.25.0 (Quinlan and Hall, 2010). Genomic regions with BLAST hits were manually inspected and the coordinates of the BLAST hits were used for annotation of pseudogenes. We also checked the presence of pseudogenes among the features annotated with Prokka package by analyzing the annotations of the adjacent genes and concluded that all of them were functional.

The search for mobile elements and phages in the genome of *Ca*. P. novymonadis was performed algorithmically in Phispy v. 2.3 (Akhter et al., 2012), as well as using database searches on the online web servers Phaster (Arndt et al., 2016) and IS Finder^5^ using *E*-value cut-off of 10^-2^.

### Analyses of Genome Sequence Properties

Files with the genome sequences and corresponding annotations for the species of interest were downloaded from the NCBI Genome database (12.12.2016). Pseudogene sequences were excluded from further analyses. Lengths of genes and intergenic regions were calculated based on the gene coordinates within GFF files containing annotation data.

For the analysis of GC content, nucleotide sequences of all genes were extracted using Artemis genome browser release v. 16.0.0 (Rutherford et al., 2000). GC content was calculated with Infoseq script from EMBOSS package v. 6.6.0.0 (Rice et al., 2000). Statistical significance of the differences in GC content, lengths of genes, and intergenic regions was tested using one-way analysis of variance (ANOVA) combined with Tukey’s honest significance test in R with *p*-value < 0.05.

Nucleotide composition by codon position, amino acid composition, and codon usage bias of protein-coding genes were analyzed using MEGA 7.0 software (Kumar et al., 2016) on the concatenated sequences of all these genes within a genome. Standard deviation of relative synonymous codon usage (RSCU) values (Sharp et al., 1986) was calculated as an integral measure of codon usage bias in a particular species. Stop codons and the two amino acids coded by only one codon (methionine and tryptophan) were excluded.

## Results and Discussion

### General Characterization of *Ca*. P. novymonadis Genome

The genome of *Ca.* P. novymonadis was assembled into six contigs with a total length of approximately 1.16 Mb (**Supplementary Table S1**), which is smaller than in free-living *Pandoraea* spp. (4.46–6.5 Mb) or in both *Polynucleobacter* spp. (1.56–2.16 Mb), but larger that in *Ca.* Kinetoplastibacterium spp. (∼0.8 Mb). The average coverage with the paired-end 100 nt Illumina HiSeq and 300 nt MiSeq reads was ∼874× and the largest contig had the length of 844,906 nt. The two shortest contigs (5,920 and 1,318 bp), containing genes for ribosomal RNA, translation factor Tu 1 and tRNAs for alanine, isoleucine, and tryptophan had approximately doubled coverage (1,555× and 1,864×, respectively) pointing to the probable duplication of these fragments in the genome. The assessment of genome assembly and annotation completeness with single-copy orthologs using BUSCO demonstrated that 147/148 (99.3%) universal genes from bacteria dataset, 216/221 (97.7%) from proteobacteria, and 529/582 (90.9%) from betaproteobacteria were present. This indicates that our assembly was complete.

Currently, there are 1,015 annotated genes, 968 of which are protein-coding. For comparison, free-living *Pandoraea* spp. have 3,960–5,342, *Polynucleobacter* spp. – 1,401 and 2,115, while *Ca.* Kinetoplastibacterium spp. only 690–732 protein-coding genes (**Supplementary Table S1**). The number of identified pseudogenes in *Ca.* P. novymonadis (13) is significantly smaller than in other species of the genus *Pandoraea* (76–361) but is comparable to that in *Ca.* Kinetoplastibacterium spp. (2–19) (**Supplementary Table S1**). Interestingly, *P. necessarius* possesses a high number of pseudogenes (269), which is apparently indicative of intense process of genome evolution and is in agreement with a recent origin of endosymbiosis in this species (Vannini et al., 2007).

No mobile elements were found in the genome of *Ca.* P. novymonadis with any of the used tools. This appears to be a consequence of genome minimization. The genome of this species has lost ∼80% of its length and protein-coding capacity compared to the genomes of its free-living *Pandoraea* spp. (**Supplementary Table S1**). We did not find statistically significant differences between the lengths of genes and intergenic regions of *Ca.* P. novymonadis compared to other *Pandoraea* spp., *Ca.* Kinetoplastibacterium spp., *Polynucleobacter* spp., *C. basilensis*, and *B. cepacia* (**Supplementary Figure S1**).

The comparison of GC content in *Ca.* P. novymonadis with that of *P. apista, P. necessarius*, and *Ca.* Kinetoplastibacterium crithidii genomes revealed significant differences both in genes and intergenic regions between *Ca.* P. novymonadis and the other analyzed species (**Supplementary Figure S2**). Interestingly, these differences were most pronounced in the genomes of trypanosomatid endosymbionts, *Ca.* P. novymonadis, and *Ca.* K. crithidii. The average GC content of the *Ca.* P. novymonadis genome (43.8%) is intermediate between that of the free-living *Pandoraea* spp. (62–65%) and *Ca.* Kinetoplastibacterium spp. (30–33%). However, it is similar to that of both endosymbiotic and free-living *Polynucleobacter* spp. (45.6 and 44.8, respectively). This pattern is also conspicuous when considering nucleotide composition in protein coding genes by codon position, with the most pronounced differences at the third position (**Supplementary Figure S3**). We found 35 genes in the *Ca.* P. novymonadis genome with the GC content higher than 56% and all these genes encode tRNAs. This is in agreement with an earlier observation that in prokaryotes the GC content of such genes does not correlate with that of the whole genome (Kawai and Maeda, 2009).

The amino acid frequencies in *Ca.* P. novymonadis differ from those in its close relatives. The most discordant ones are for alanine, isoleucine, and lysine (**Supplementary Figure S4**). As with the nucleotide composition, the amino acids frequencies in this species are intermediate between those of other *Pandoraea* spp. and *Ca.* Kinetoplastibacterium spp. and appear most similar to those in *Polynucleobacter* spp.

In agreement with the previously described general trend, the codon usage bias in analyzed species correlated with genomic GC content (Sharp et al., 2005). This relationship was represented by a sideways parabola with the vertex (i.e., lowest value of RSCU standard deviation) situated at about 50% GC: further from the equilibrium nucleotide frequencies, the more pronounced was the bias. Most of the Alcaligenaceae and Burkholderiaceae species fitted this parabolic curve. Three notable exceptions were *Ca.* P. novymonadis (possessing the least prominent codon usage bias) and the two *Polynucleobacter* spp. (**Supplementary Figure S5**). It was previously proposed that species under selection for rapid growth have stronger codon usage bias (Sharp et al., 2005, 2010). However, this is not the case here. In terms of growth rate, the outliers *Ca.* P. novymonadis and *P. necessarius* do not differ much from *Ca.* Kinetoplastibacterium spp. fitting to the trend, since all these bacteria are endosymbionts. An alternative explanation appears to be more plausible: the bacteria that have to switch gene expression from time to time (usually owing to the changing environment) have a stronger bias as compared to those living in stable conditions (Botzman and Margalit, 2011). Although *Ca.* Kinetoplastibacterium spp. are endosymbionts, their close interactions with the host, reflected by a tight coordination of their cell divisions, may lead to similar switches. As for *Ca.* P. novymonadis, its relationship with the host cell seems to be more relaxed (Kostygov et al., 2016) and apparently does not require complex gene expression.

Synteny analysis with free-living *Pandoraea* spp. demonstrated that 62–69% of “anchors” (pairwise alignments) in *Ca.* P. novymonadis genome are located within synteny blocks with maximal values observed for *P. faecigallinarum* and *P. vervacti* (Supplementary Table S2). The fact that the majority of the synteny blocks are inverted (15/24 and 11/21 for *P. faecigallinarum* and *P. vervacti*, respectively), reflects a relatively long evolutionary distances between these species and *Ca.* P. novymonadis. The pairwise synteny between *Ca.* P. novymonadis and the genomes of other *Pandoraea* spp. available in GenBank is presented in **Supplementary Figure S6**. This analysis demonstrated the reduction of the *Ca.* P. novymonadis genome compared to those of free-living *Pandoraea* spp. and a high number of genome rearrangements occurring in the evolution of this endosymbiotic bacterium.

Thus, sequencing and annotation of the *Ca.* P. novymonadis genome revealed several features characteristic for other endosymbiotic bacteria: reduced size, massive gene losses, and decrease in GC content as compared to the genomes of its free-living relatives (Boscaro et al., 2017). Taken together, *Ca.* P. novymonadis is closer to *P. necessarius* than to *Ca.* Kinetoplastibacterium spp.

### Phylogenomic Analysis

**FIGURE 1:**
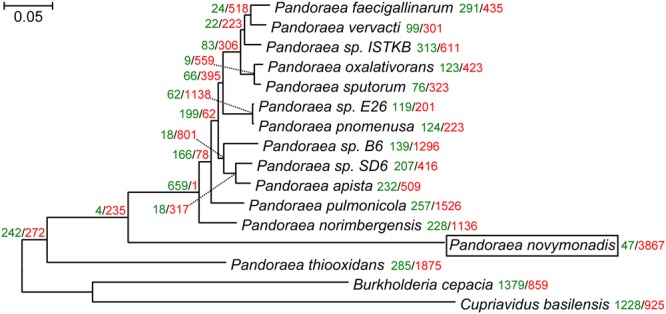
The maximum-likelihood phylogenetic tree of the genus *Pandoraea* reconstructed on a concatenated alignment of 119 conserved proteins with gains and losses mapped using Dollo parsimony algorithm. All branches have 100% bootstrap support and posterior probability of 1.0. The scale bar denotes the number of substitutions per site. The tree was rooted with *Cupriavidus basilensis* and *Burkholderia cepacia.* Gene gains and losses are marked with green and red color, respectively. At all the leaves and most of the nodes within the *Pandoraea* clade gene losses dominate. This is especially pronounced in the endosymbiotic *Ca.* P. novymonadis, which gained only 47 and lost 3,867 OGs.

The maximum likelihood and Bayesian trees inferred using the supermatrix containing 119 genes displayed identical topologies with all branches having maximal bootstrap percentage and posterior probabilities. Previous reconstruction, based on the 16S rRNA gene sequences, placed *Ca.* P. novymonadis in the very crown of the *Pandoraea* clade, though with a low support (Kostygov et al., 2016). However, the results presented here, which are based on much more extensive dataset, demonstrate this species to be an early branch diverged next to *P. thiooxidans* (**Figure 1**). The same position of *Ca.* P. novymonadis could be observed in analyses using either 556 genes supermatrix, or concatenated 16S rRNA and 23S rRNA genes, or a popular bacterial marker, *gyrB* (data not shown). As compared to other *Pandoraea* spp., the species under study demonstrated significantly longer branch (**Figure 1**). This is related to multiple amino acid substitutions in conserved sites and may be explained by fast adaptive evolution of this species. However, in comparison with the outgroups *B. cepacia* and *C. basilensis*, the branch of *Ca.* P. novymonadis does not appear to be uniquely long (**Figure 1**).

### Analysis of Protein Orthologous Groups

We performed OrthoFinder analysis on a dataset of 23 annotated bacterial genomes (**Supplementary Table S1**). This resulted in 12,248 OGs, of which 5,437 contained only one protein. Similarly to the *Ca.* Kinetoplastibacterium spp. (Alves et al., 2013b), the genome of *Ca.* P. novymonadis is minimized and the vast majority of genes are single-copy: we found only five OGs containing two proteins with the sequence identity varying from 36 to 96%. These proteins were annotated as ATP-dependent RNA helicase, NADP^+^ reductase, BolA family transcriptional regulator, alanine-tRNA ligase, and threonine synthase. According to our analysis, ATP-dependent RNA helicase and NADP^+^ reductase were also duplicated in the genomes of several *Ca.* Kinetoplastibacterium spp. This situation is drastically different from that observed in the free-living *Pandoraea* spp., which have a substantially higher number of OGs containing two or more genes (e.g., 338 OGs in *P. apista* and 324 in *P. pnomenusa*).

We mapped gene family gains and losses on the phylogenomic tree (**Figure 1**). Gene loss is a predominant trend for all the leaves and most of the nodes within the *Pandoraea* clade. It is especially pronounced in the endosymbiotic *Ca.* P. novymonadis, which gained only 47 and lost 3,867 OGs. We used a sensitive HHpred tool attempting to illuminate functions of the proteins within OGs specific for *Ca.* P. novymonadis (Supplementary Table S3). Only 9 out of 47 proteins could be annotated using an *E*-value cut-off of 1. The following putative domains were identified: histidine kinase-like ATPase, cytoplasmic E component of the type III secretion system needle, myristoyl-CoA:protein *N*-myristoyltransferase, and carbohydrate binding domain.

We grouped gene annotations for the 3,867 OGs lost in *Ca.* P. novymonadis according to the KO system. Most of them belong to the following categories: “environmental information processing,” “amino acid metabolism,” “carbohydrate metabolism,” “genetic information processing,” “xenobiotics biodegradation,” and “energy metabolism” (**Supplementary Figure S7**). Out of 3,867 OGs, 1,273 were uniquely lost in *Ca*. P. novymonadis. The composition of functional categories assigned to the proteins within these OGs according to the KO system is similar to that assigned to all 3,867 OGs lost in *Ca*. P. novymonadis. However, the proportion of proteins belonging to the categories “genetic information processing,” “energy metabolism,” and “lipid metabolism” is increased in case of the annotations of OGs uniquely lost in *Ca*. P. novymonadis. The largest portion of OGs lost in *Ca*. P. novymonadis belong to the functional category “environmental information processing,” and more specifically “ATP-binding cassette transporters (ABC transporters).” *Ca*. P. novymonadis has lost many members of this protein family as compared to free-living *Pandoraea* spp.: mineral and organic ion transporters (e.g., for sulfate, nitrate, taurine, molybdate), monosaccharide transporters (e.g., for glycerol-3-phosphate), phosphate and amino acid transporters (e.g., for phosphate, phosphonate, glutamate, aspartate, cystine, urea, D-methionine), and transporters for glutathione and lipooligosaccharides.

Interestingly, there were no OGs uniquely shared between *Ca. P. novymonadis* and either of the endosymbiotic bacterial species investigated here (**Supplementary Figure S8**).

### Lipid Metabolism

We identified a full set of enzymes essential for the type-II fatty acid synthesis (FAS) in *Ca.* P. novymonadis and other *Pandoraea* spp., *Ca.* Kinetoplastibacterium spp., *C. basilensis, B. cepacia*, and *Polynucleobacter* spp. (Supplementary Table S4). Acetyl-CoA carboxylase, the starting enzyme of the type-II FAS, in bacteria is composed of several polypeptides encoded by four distinct genes: *accA, accB, accC*, and *accD*. The *accB* and *accC* genes in *Ca.* P. novymonadis are located adjacent to each other and belong to the same operon, similarly to the situation observed in *Escherichia coli* (Janssen and Steinbuchel, 2014). FabF and FabH, 3-ketoacyl-acyl-carrier-protein (ACP) synthases II and III, which catalyze the formation of 3-ketoacyl-ACP by condensation of fatty acyl-ACP with malonyl-ACP, are present, while 3-ketoacyl-ACP synthase II (FabB) is absent in all the analyzed genomes, except for *P. oxalativorans* and *P. vervacti*. FabB participates in the synthesis of unsaturated fatty acids (FAs), catalyzing the condensation of *cis*-3-decenoyl-ACP (formed by the FabA catalyzed reaction), *cis*-5-dodecenoyl-ACP, and *cis*-7-tetradecenoyl-ACP with malonyl-ACP (Feng and Cronan, 2009). 3-Hydroxydecanoyl-ACP dehydratase/isomerase (FabA), another key player in the synthesis of unsaturated FA is also missing from the analyzed genomes. Interestingly, *C. basilensis* possesses three different enoyl-ACP reductases, catalyzing the last step of the elongation cycle in the synthesis of FA: FabI, FabK, and FabV (Massengo-Tiassé and Cronan, 2009). *Ca.* P. novymonadis, *Ca.* Kinetoplastibacterium spp., and *Polynucleobacter* spp. have only FabI-encoding gene. The majority of the free-living *Pandoraea* spp. retain only FabV, while B. *cepacia, P. norimbergensis, P. oxalativorans, P. pulmonicola*, and *P. thioxidans* retain FabK along with FabV. The physiological rationale for the presence of multiple enoyl-ACP reductases is poorly understood (Zhu et al., 2013).

All *Pandoraea* spp., *Polynucleobacter* spp., *C. basilensis*, and *B. cepacia* are able to synthesize cardiolipin, phosphatidylethanolamine, and phosphatidyl-L-serine, important components of the bacterial membranes (Supplementary Table S5). In all bacteria analyzed, the end product of the FA biosynthesis, acyl-ACP, can be activated with an inorganic phosphate group by the action of the PlsX component of the PlsX/PlsY/PlsC acyltransferase system, leading to acyl-phosphate, which is subsequently added to glycerol-3-phosphate by the action of the PlsY component (Janssen and Steinbuchel, 2014). The next steps to synthesize diacylglycerol-3-phosphate and cytosine diphosphate diacylglycerol (CDP-diacylglycerol) are performed by 1-acyl-sn-glycerol-3-phosphate acyltransferase (PlsC) and phosphatidate cytidylyltransferase (CdsA). CDP-diacylglycerol is the intermediate which is then used for the formation of cardiolipin, phosphatidyl-L-serine, and phosphatidylethanolamine by cardiolipin synthase, CDP-diacylglycerol-serine *O*-phosphatidyltransferase, and phosphatidylserine decarboxylase, respectively. All *Ca*. Kinetoplastibacterium spp. lack the capacity to synthesize cardiolipin, while *Ca*. K. galatii, *Ca.* K. oncopeltii, and *Ca.* K. blastocrithidii are not able to produce any of the membrane lipids mentioned above.

Interestingly, no lipases and esterases could be detected in the genome of *Ca*. P. novymonadis even with the *E*-value cut-off of 10^-10^. We found proteins belonging to the family VI of bacterial lipolytic enzymes in all *Ca*. Kinetoplastibacterium spp. and in *P. necessarius* (Arpigny and Jaeger, 1999). The lipases and esterases belonging to the families I, IV, V, and VI are readily identifiable in the genomes of *C. basilensis* and *B. cepacia*, as well in the free-living *Pandoraea* spp., which in addition possess proteins belonging to the family VII of the lipolytic enzymes.

Importantly, all endosymbionts of trypanosomatids, including *Ca.* P. novymonadis, are unable to oxidize FAs since all the enzymes required for β-oxidation are missing, similarly to the situation observed in bacterial endosymbionts of insects (Zientz et al., 2004).

### Carbon Metabolism

All species analyzed in this work preserve enzymes for glycolysis and the central (non-oxidative) part of the pentose phosphate pathway (**Supplementary Figure S9**). However, only the free-living *Pandoraea* spp. have hexokinase and, thus, are able to utilize glucose. In contrast to the endosymbiotic bacteria, they also can use classic and alternative (i.e., non-phosphorylated) variants of the Entner–Doudoroff pathway. Interestingly, only *P. thiooxidans* possesses phosphofructokinase converting fructose-6-phosphate into fructose 1,6-bisphosphate. Other species must use a bypass through the pentose phosphate pathway for hexose catabolism. Fructose 1,6-bisphosphatase, the enzyme catalyzing the reverse reaction, is present in all studied species suggesting its importance for anabolic processes, in particular, gluconeogenesis.

We were unable to trace the carbon source that *Ca.* Kinetoplastibacterium spp. utilize instead of glucose. However, for *Ca.* P. novymonadis and *P. necessarius* this appears to be fructose. Similarly to the situation with glucose, there is no typical phosphorylating enzyme, i.e., fructokinase (it is also absent from all other *Pandoraea* spp.). In all these species we identified the three cytoplasmic components of phosphotransferase system (PTS), namely phosphoenolpyruvate (PEP)-protein phosphotransferase (PTS-EI), histidine phosphocarrier protein (HPr), and PTS system fructose-specific EIIA component (PTS-EIIA^Fru^). The main function of PTS is a concomitant transfer of sugars inside the cell and their phosphorylation (Saier, 2015). In addition to the three proteins mentioned above, the fully functional PTS must also contain juxtamembrane permease PTS-EIIB and transmembrane PTS-EIIC (sometimes along with PTS-EIID). The phosphate from PEP is successively transferred to PTS-EI, then to HPr, PTS-EIIA, PTS-EIIB, and then to sugar (Saier, 2015). Numerous proteobacteria possess incomplete PTS-lacking EIIB and EIIC components. Such PTSs were proposed to have only regulatory functions (Deutscher et al., 2014). We hypothesize that the incomplete fructose-specific PTS may be used for phosphorylation of fructose. Indeed, in addition to the abovementioned lack of fructokinase, *Ca.* P. novymonadis also does not have pyruvate kinase, the key enzyme for the production of ATP from PEP at the end of glycolysis. Meanwhile, PTS using PEP as a phosphate donor could substitute this missing link. The lack of hexokinase and fructokinase along with the presence of PTS was also documented in obligate intracellular bacteria of insects (Zientz et al., 2004).

The complete tricarboxylic acid (TCA) cycle is present in all considered bacteria except *Ca.* Kinetoplastibacterium spp., which possess enzymes for two consecutive steps of this cycle: transformation of 2-oxoglutarate to succinyl-CoA and then to succinate. These steps may be preserved because succinyl-CoA is required for lysine biosynthesis. In addition, these bacteria possess malate dehydrogenase interconverting malate and oxaloacetate.

In addition to the TCA cycle, the free-living *Pandoraea* spp. also have the complete glyoxylate pathway, enabling usage of short-chain compounds as a carbon source. Endosymbiotic bacteria in their stable environment do not need such capability. Intriguingly, *P. necessarius* has malate synthase interconverting glyoxylate and malate, whereas other enzymes of this cycle are absent from its genome.

### Amino Acid Metabolism

**FIGURE 2:**
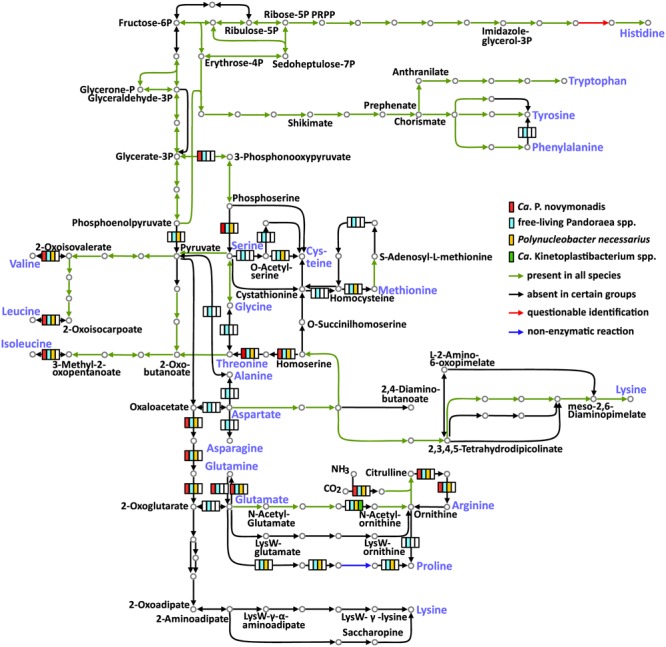
Amino acid metabolism in *Ca.* P. novymonadis, free-living *Pandoraea* spp., *Polynucleobacter necessarius*, and *Ca.* Kinetoplastibacterium spp. The 20 standard amino acids are shown in violet.

The free-living *Pandoraea* spp. are able to synthesize all 20 amino acids. Meanwhile, the three groups of endosymbionts considered here (*Ca. P. novymonadis, P. necessarius*, and *Kinetoplastibacterium* spp.) demonstrate different phases of gradual loss of those capabilities (**Figure 2** and Supplementary Table S6). This process starts with the loss of the pathways for the synthesis of the non-essential amino acids such as alanine, asparagine, and aspartate, a situation observed in the evolutionary young endosymbiont *P. necessarius. Ca.* P. novymonadis is unable to synthesize three additional amino acids: cysteine, methionine, and proline. *Ca.* Kinetoplastibacterium spp. exhibit the most advanced state, lacking enzymes for the synthesis of 13 amino acids (**Figure 2**). As judged from previous studies, the metabolic pathways of these endosymbionts and their hosts are interlaced and, for most of the amino acids, the enzymes missing in the bacterium can be substituted by those of the trypanosomatid (Alves et al., 2013a; Alves, 2017). Although the metabolism of *N. esmeraldas* has not been studied yet, it is likely similar to that of its relatives – trypanosomatids of the subfamily Leishmaniinae. This group of flagellates is auxotrophic for arginine, histidine, isoleucine, leucine, phenylalanine, serine, tryptophan, tyrosine, and valine (Opperdoes et al., 2016). Therefore, it is not surprising that *Ca.* P. novymonadis retained the ability to synthesize them. In return, *N. esmeraldas* may provide the six amino acids, which its symbiont is unable to produce.

In addition to losing the ability to synthesize particular amino acids, the endosymbionts are devoid of some biochemical bypasses. Thus, phenylalanine-4-hydroxylase, converting phenylalanine to tyrosine, is present in free-living *Pandoraea* spp., but absent in all endosymbionts analyzed here. The same concerns arginase, the enzyme transforming arginine to ornithine (**Figure 2**).

Histidinol-phosphate phosphatase (HPpase), responsible for the penultimate step of histidine biosynthesis, was not found by BlastKOALA in any of the analyzed genomes. Nevertheless, HPpases are present in GenBank genome annotations of all free-living *Pandoraea* spp. Homologous proteins in *Polynucleobacter* spp. and *Ca.* Kinetoplastibacterium spp. are annotated as inositol monophosphatases. The same result was obtained for *Ca.* P. novymonadis in Prokka annotation. It is known that inositol-monophosphatase-like enzymes may exhibit histidinol-phosphatase activity (Mormann et al., 2006; Petersen et al., 2010; Nourbakhsh et al., 2014). Of note, none of the bacteria analyzed here has other enzymes of inositol metabolism, so it is unlikely that the protein in question is an inositol monophosphatase. Thus, we argue that all analyzed species possess divergent histidinol-phosphatases.

### Urea Cycle/Polyamine Synthesis

**FIGURE 3:**
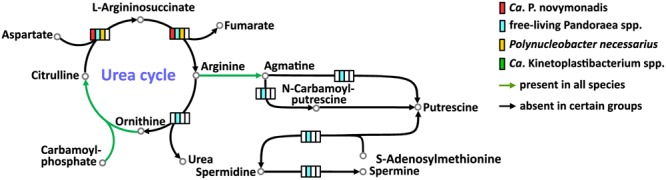
Urea cycle and polyamine synthesis in *Ca.* P. novymonadis, free-living *Pandoraea* spp., *Polynucleobacter necessarius*, and *Ca.* Kinetoplastibacterium spp.

All free-living *Pandoraea* spp. have complete set of enzymes for the urea cycle and synthesis of important polyamines. *Ca.* P. novymonadis and *P. necessarius* lack arginase, while preserving ornithine carbamoyltransferase, argininosuccinate synthase, and argininosuccinate lyase (**Figure 3**). They also possess arginine decarboxylase converting arginine to agmatine, the first intermediate in the synthesis of polyamines, but for the rest of this pathway these bacteria apparently rely on their respective hosts. *Ca.* Kinetoplastibacterium spp. showed the most reduced state with only two enzymes remaining in their arsenal: carbamoyltransferase and arginine decarboxylase (**Figure 3**).

### Vitamins and Cofactors

All bacteria analyzed here are able to synthesize a number of porphyrins, including heme, an essential compound for most trypanosomatids (Kořený et al., 2012). The free-living *Pandoraea* spp., *Ca*. P. novymonadis and *P. necessarius*, are prototrophic for all vitamins. As for *Ca*. Kinetoplastibacterium spp., their metabolism of vitamins was previously scrutinized by others (Klein et al., 2013). It has been demonstrated that in contrast to the rest of bacteria considered here, they are unable to synthesize thiamine, nicotinic acid, and biotin, which are apparently acquired by the trypanosomatid host from the insect’s gut content. All enzymes needed to produce folic acid, vitamin B6, and riboflavin essential for the trypanosomatid host are encoded in the genomes of *Ca*. Kinetoplastibacterium spp., but the pathway of pantothenic acid biosynthesis is interrupted at the very end (Klein et al., 2013). The missing enzyme (ketopantoate reductase) is encoded in the genome of the trypanosomatid host, thus representing an example of deep integration of metabolic pathways in this symbiotic association.

## Conclusion

Here, we sequenced and analyzed the genome of *Ca.* P. novymonadis, the bacterial endosymbiont of the trypanosomatid *N. esmeraldas*. To better understand the evolution and biology of this bacterium, we compared its genome to those of related prokaryotes, namely the free-living *Pandoraea* spp., two sister *Polynucleobacter* spp., from which one is free-living and the other is endosymbiotic, as well as with *Ca*. Kinetoplastibacterium spp., which are the only other known endosymbionts of trypanosomatids. The genome of *Ca.* P. novymonadis revealed all hallmarks of an endosymbiont genome: size reduction, massive gene losses, decreased GC content, and lowered codon usage bias. At the same time, this genome preserves main metabolic pathways, including biosynthesis of vitamins and heme, essential for the trypanosomatid host. The bacterium does not produce some amino acids, which are likely provided by the host, but retains the ability to synthesize those, for which the trypanosomatid is auxotrophic.

Our data allow first comparative analysis of the endosymbionts of trypanosomatids and strongly indicate that their evolution followed different scenarios, reflected by the fact that they do not have uniquely shared traits. Importantly, from the perspective of both its general genomic features and metabolism, *Ca.* P. novymonadis is closer to the ciliate-dwelling *P. necessarius*, which belongs to the same family Burkholderiaceae, than to *Ca*. Kinetoplastibacterium spp., the only other known endosymbionts of trypanosomatids.

Previously, we proposed that the endosymbiosis between *Ca.* P. novymonadis and *N. esmeraldas* was established relatively recently (Kostygov et al., 2016). This opinion was based on the phylogenetic position of the bacterium and seemingly unsophisticated relationships in this symbiotic association. However, the phylogenomic analysis presented here demonstrates that the endosymbiont diverged earlier than as inferred from its 16S rRNA gene. As judged from its genomic characteristics, *Ca.* P. novymonadis has already passed the intensive period of host adaptation, which can still be observed in *P. necessarius*, the best candidate for a recent endosymbiosis. As judged by the extent of the overall genome reduction and the loss of particular metabolic abilities correlating with the increasing dependence of the symbiont on its host, *Ca*. P. novymonadis occupies an intermediate position *P. necessarius* and *Ca*. Kinetoplastibacterium spp. We conclude that the relationship between *Ca.* P. novymonadis and *N. esmeraldas* is already well-established, although not as fine-tuned as in the case of related flagellates of the family Strigomonadinae and their endosymbionts.

## Author Contributions

VY and JL jointly conceived the study. AK and AB contributed equally to this work: participated in the design of the study, the analysis and interpretation of data, and the manuscript writing. AN and PF conducted genome assembly and curated annotation, and contributed to the interpretation of data. DT established and analyzed the aposymbiotic strain of *N. esmeraldas*. VY, AK, and JL revised and corrected the manuscript. All authors read and approved the final manuscript.

## Conflict of Interest Statement

The authors declare that the research was conducted in the absence of any commercial or financial relationships that could be construed as a potential conflict of interest.

## Footnotes

1 https://sourceforge.net/projects/bbtools/

2 http://www.bioinformatics.babraham.ac.uk/projects/fastqc/

3 https://academic.oup.com/bioinformatics/article/31/20/3350/196114/Bandage-interactive-visualization-of-de-novo

4 https://cran.r-project.org/web/packages/UpSetR/

5 http://phaster.ca and https://www-is.biotoul.fr

## Supplementary Material

FIGURE S1 Graphs showing length distribution for genomic features in *Ca.* P. novymonadis and other bacteria with various lifestyles. **(A,C)** Distribution of gene lengths; **(B,D)** distribution of the lengths of intergenic regions. Pnov, *Ca.* P. novymonadis; Bcep, *B. cepacia*; Cbas, *C. basilensis*; Papi, *Pandoraea apista*; Pfae, *Pandoraea faecigallinarum*; Pnor, *Pandoraea norimbergensis*; Poxa, *Pandoraea oxalativorans*; Ppno, *Pandoraea pnomenusa*; Ppul, *Pandoraea pulmonicola*; Pspu, *Pandoraea sputorum*; Pthi, *Pandoraea thiooxidans*; Pver, *Pandoraea vervacti*; Kbla, *Ca.* Kinetoplastibacterium blastocrithidii; Kcri, *Ca.* Kinetoplastibacterium crithidii; Kdes, *Ca.* Kinetoplastibacterium desouzai; Kgal, *Ca.* Kinetoplastibacterium galatii; Konc, *Ca.* Kinetoplastibacterium oncopeltii; Pasy, *Polynucleobacter asymbioticus*; and Pnec, *Polynucleobacter necessarius*.

FIGURE S2 Graphs showing GC content distribution for genomic features in *Ca.* P. novymonadis and other bacteria with various lifestyles: solid lines for genes and dotted lines for intergenic regions. Abbreviations of bacterial species names are as in **Supplementary Figure S1**; “ir” stands for intergenic regions.

FIGURE S3 Nucleotide composition of protein-coding genes by codon positions. Abbreviations of bacterial species names are as in **Supplementary Figure S1**; nucleotides in a particular codon position are denoted below the figure.

FIGURE S4 Amino acid frequencies. Abbreviations of bacterial species names are as in **Supplementary Figure S1**.

FIGURE S5 Relationship of codon usage bias and GC content in protein coding genes averaged over the whole genome. The dotted line represents a sideways parabola fitting the distribution of values. Akas, *Advenella kashmirensis*; Tequ, *Taylorella equigenitalis*; Axyl, *Achromobacter xylosoxidans*; Aspa, *Achromobacter spanius*; Bbro, *Bordetella bronchiseptica*; Bhol, *Bordetella holmesii*; Pnoe, *Pusillimonas noertemannii*; Pind, *Pelistega indica*; Oure, *Oligella urethralis*; Lisp, *Limnobacter* sp. CACIAM 66H1; Ggig, *Ca*. Glomeribacter gigasporarum; Csor, *Caballeronia sordidicola*. All other abbreviations of bacterial species names are as in **Supplementary Figure S1**.

FIGURE S6 Schematic representation of two-way synteny between *Ca.* P. novymonadis and other *Pandoraea* spp. with sequenced genomes. The four longest *Ca.* P. novymonadis contigs are colored according to the legend. Only scaffolds with synteny blocks are shown. Direct synteny blocks are displayed in red, inverted ones – in green. The contigs are drawn proportionately to their actual length. The genomes of *Pandoraea* spp. shown on the figure are fully assembled to the level of circular chromosomes depicted as the longest colored bars. For some species the shorter colored bars representing plasmids are shown in addition to the chromosomal scaffolds.

FIGURE S7 A pie chart showing gene annotations for 3,867 OGs lost in *Ca.* P. novymonadis (Pnov) categorized according to the KEGG Orthology system. *Pandoraea pnomenusa* (Ppno), *Pandoraea norimbergensis* (Pnor), and *Pandoraea vervacti* (Pver) are shown for comparison.

FIGURE S8 Analysis of OGs sharing between free-living *Pandoraea* spp., *Ca.* Pandoraea novymonadis, *Ca.* Kinetoplastibacterium spp., *Polynucleobacter* spp., *Burkholderia cepacia*, and *Cupriavidus basilensis*. OGs were categorized according to their presence in the analyzed species. Depicted bars indicate number of OGs that are unique or shared among the genomes of the organisms listed, as indicated by the black dots.

FIGURE S9 Carbon metabolism in *Ca.* P. novymonadis, free-living *Pandoraea* spp., *Polynucleobacter necessarius*, and *Ca.* Kinetoplastibacterium spp.

TABLE S1 Genomic characteristics of species used in analyses.
