# Supplementary figures and images for: Genome of Ca. Pandoraea novymonadis, an Endosymbiotic Bacterium of the Trypanosomatid Novymonas esmeraldas
Source: Front Microbiol. 2017 Oct 4;8:1940. doi: 10.3389/fmicb.2017.01940 (PMC5632650; doi:10.3389/fmicb.2017.01940)

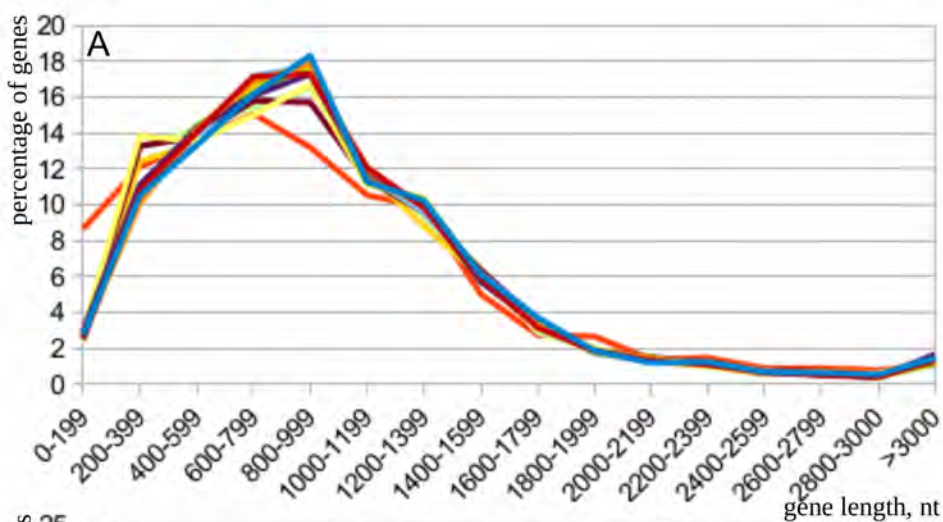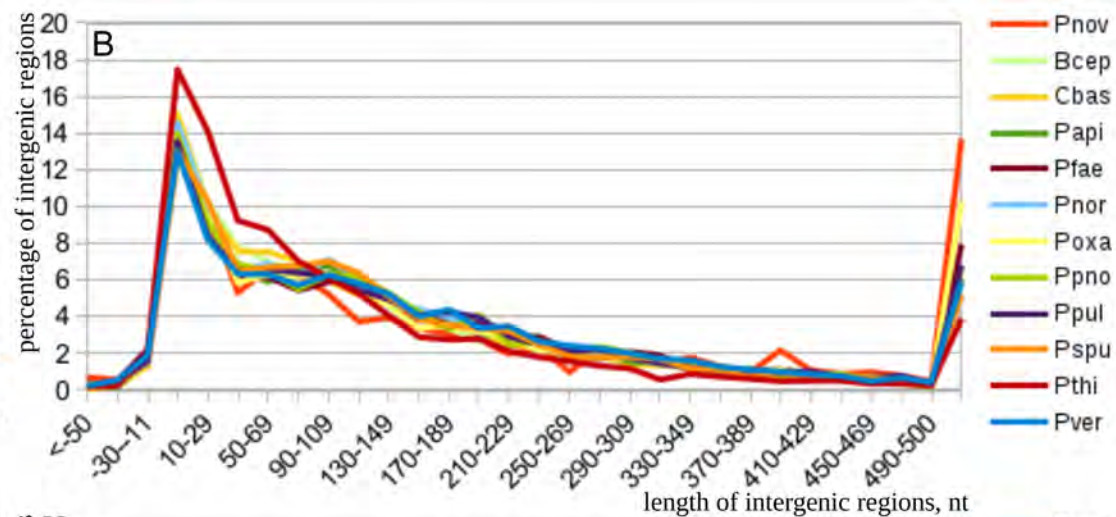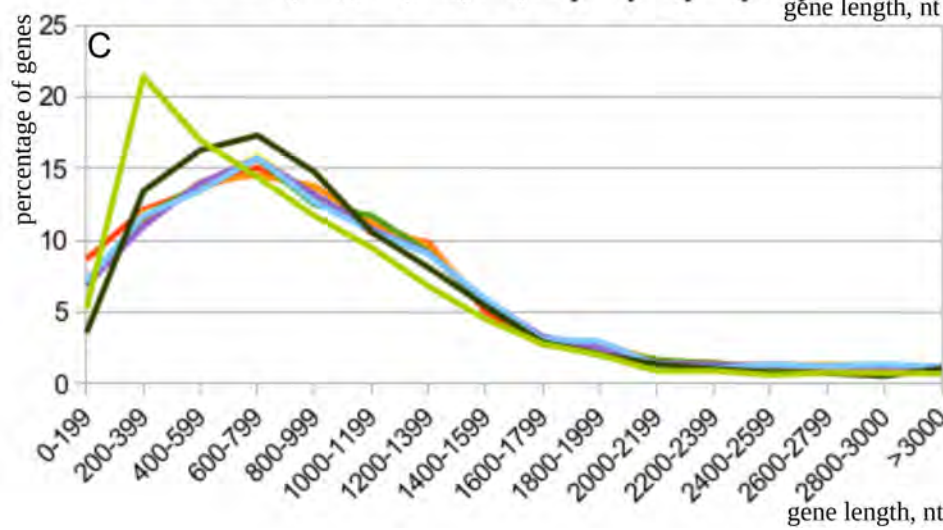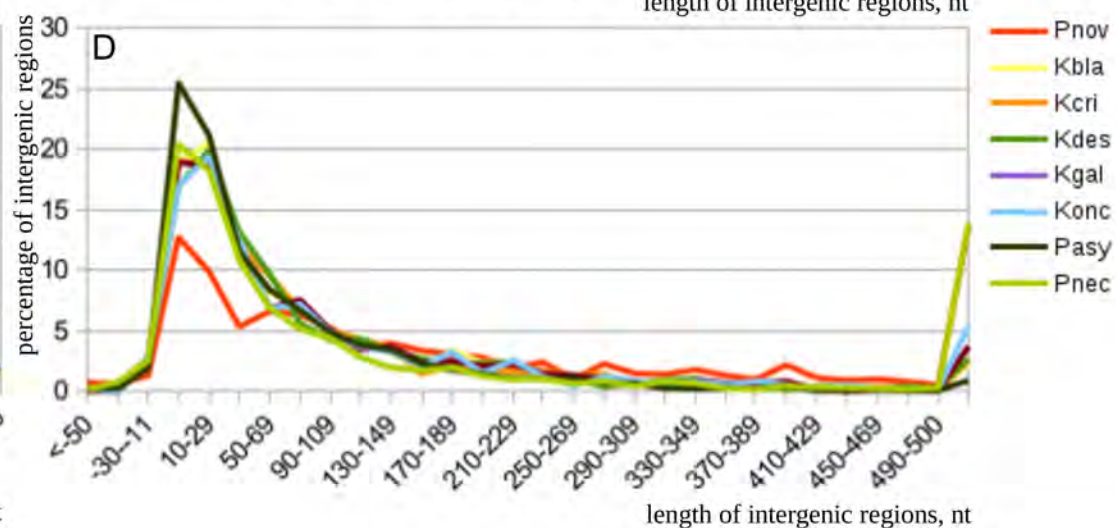

Supplement: FIGURE S1 — Graphs showing length distribution for genomic features in Ca. P. novymonadis and other bacteria with various lifestyles. (A,C) Distribution of gene lengths; (B,D) distribution of the lengths of intergenic regions. Pnov, Ca. P. novymonadis; Bcep, B. cepacia; Cbas, C. basilensis; Papi, Pandoraea apista; Pfae, Pandoraea faecigallinarum; Pnor, Pandoraea norimbergensis; Poxa, Pandoraea oxalativorans; Ppno, Pandoraea pnomenusa; Ppul, Pandoraea pulmonicola; Pspu, Pandoraea sputorum; Pthi, Pandoraea thiooxidans; Pver, Pandoraea vervacti; Kbla, Ca. Kinetoplastibacterium blastocrithidii; Kcri, Ca. Kinetoplastibacterium crithidii; Kdes, Ca. Kinetoplastibacterium desouzai; Kgal, Ca. Kinetoplastibacterium galatii; Konc, Ca. Kinetoplastibacterium oncopeltii; Pasy, Polynucleobacter asymbioticus; and Pnec, Polynucleobacter necessarius. [file Image_1.PDF]

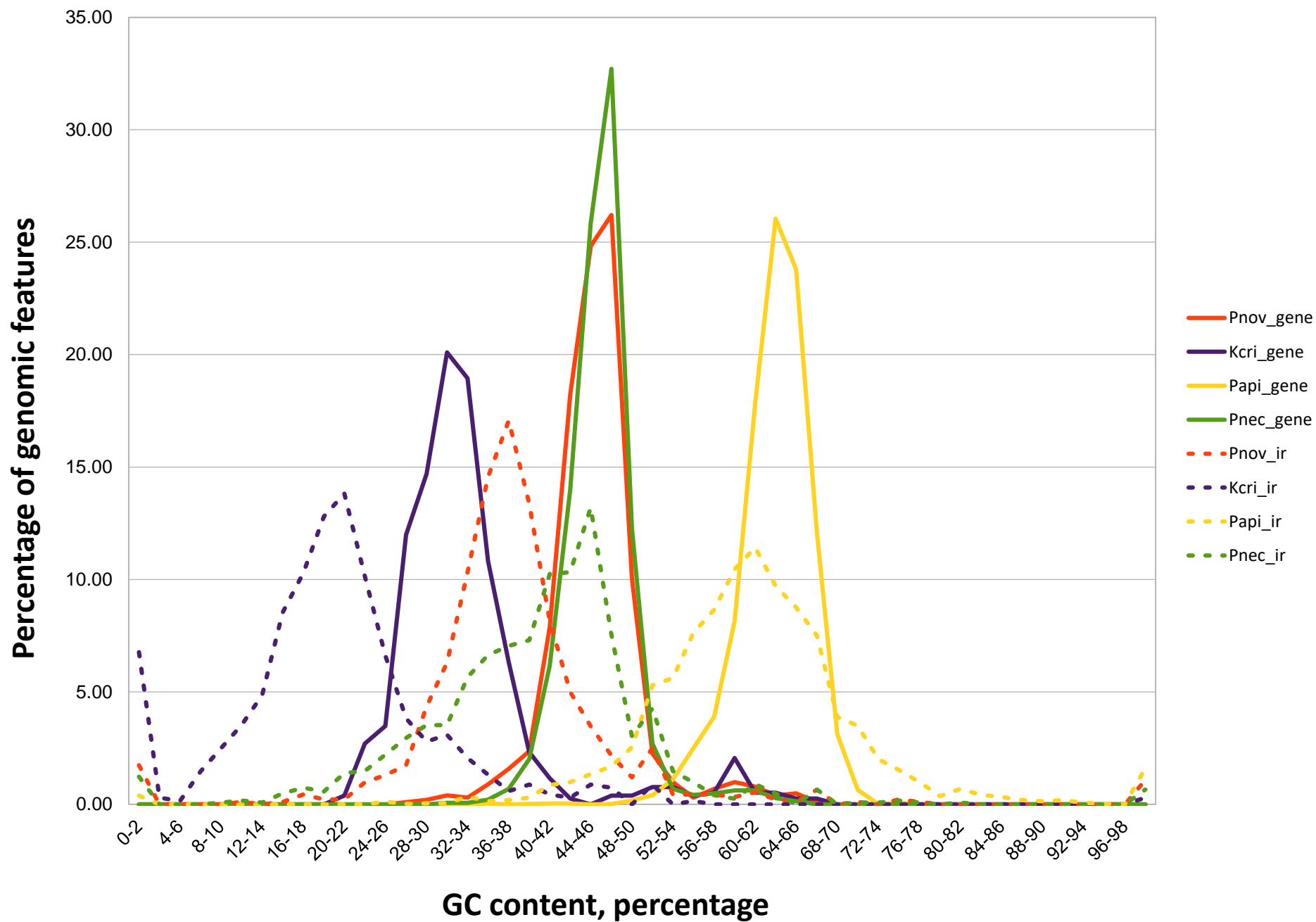

Supplement: FIGURE S2 — Graphs showing GC content distribution for genomic features in Ca. P. novymonadis and other bacteria with various lifestyles: solid lines for genes and dotted lines for intergenic regions. Abbreviations of bacterial species names are as in Supplementary Figure S1; “ir” stands for intergenic regions. [file Image_2.pdf]

Nucleotide composition by codon position

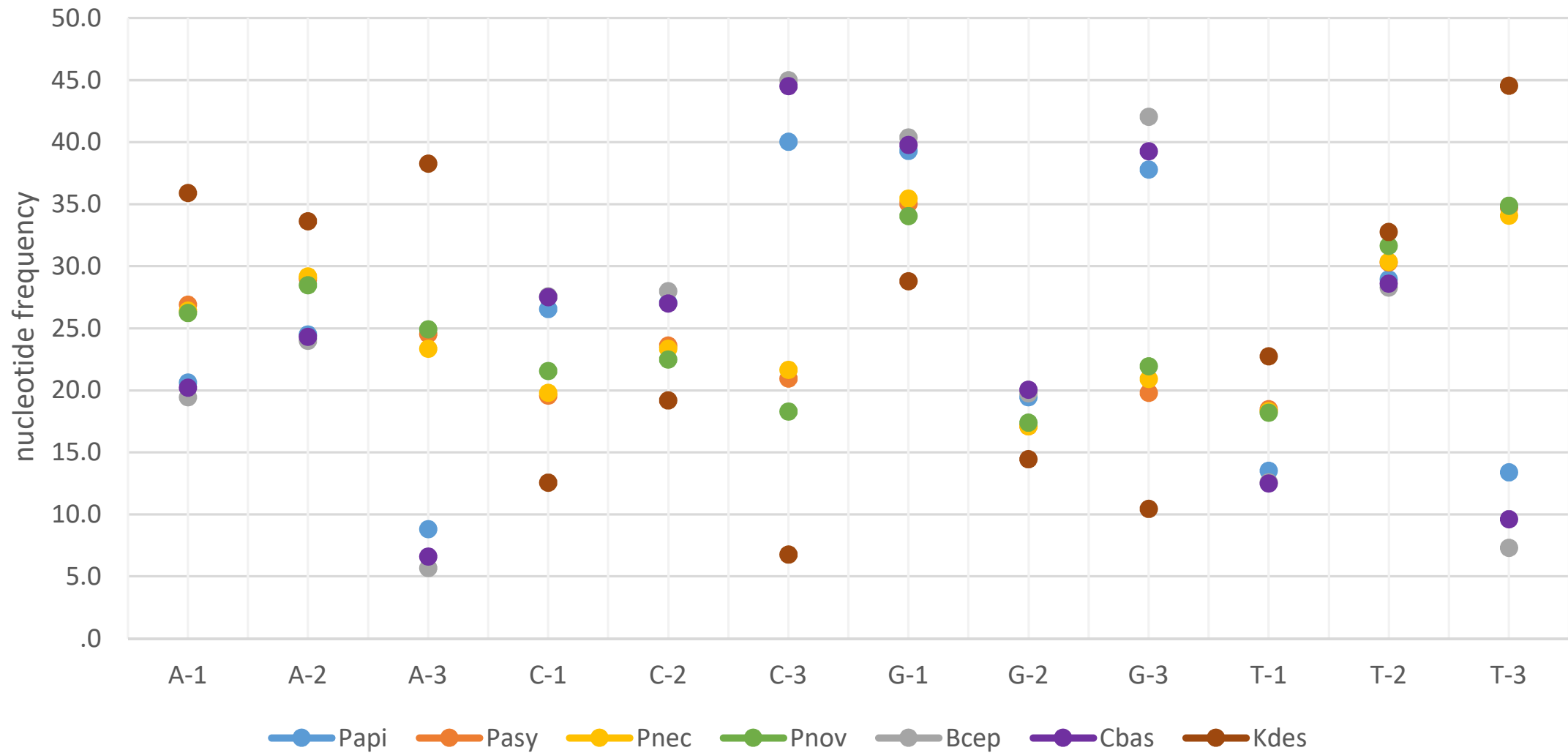

Supplement: FIGURE S3 — Nucleotide composition of protein-coding genes by codon positions. Abbreviations of bacterial species names are as in Supplementary Figure S1; nucleotides in a particular codon position are denoted below the figure. [file Image_3.PDF]

Amino acid composition, %

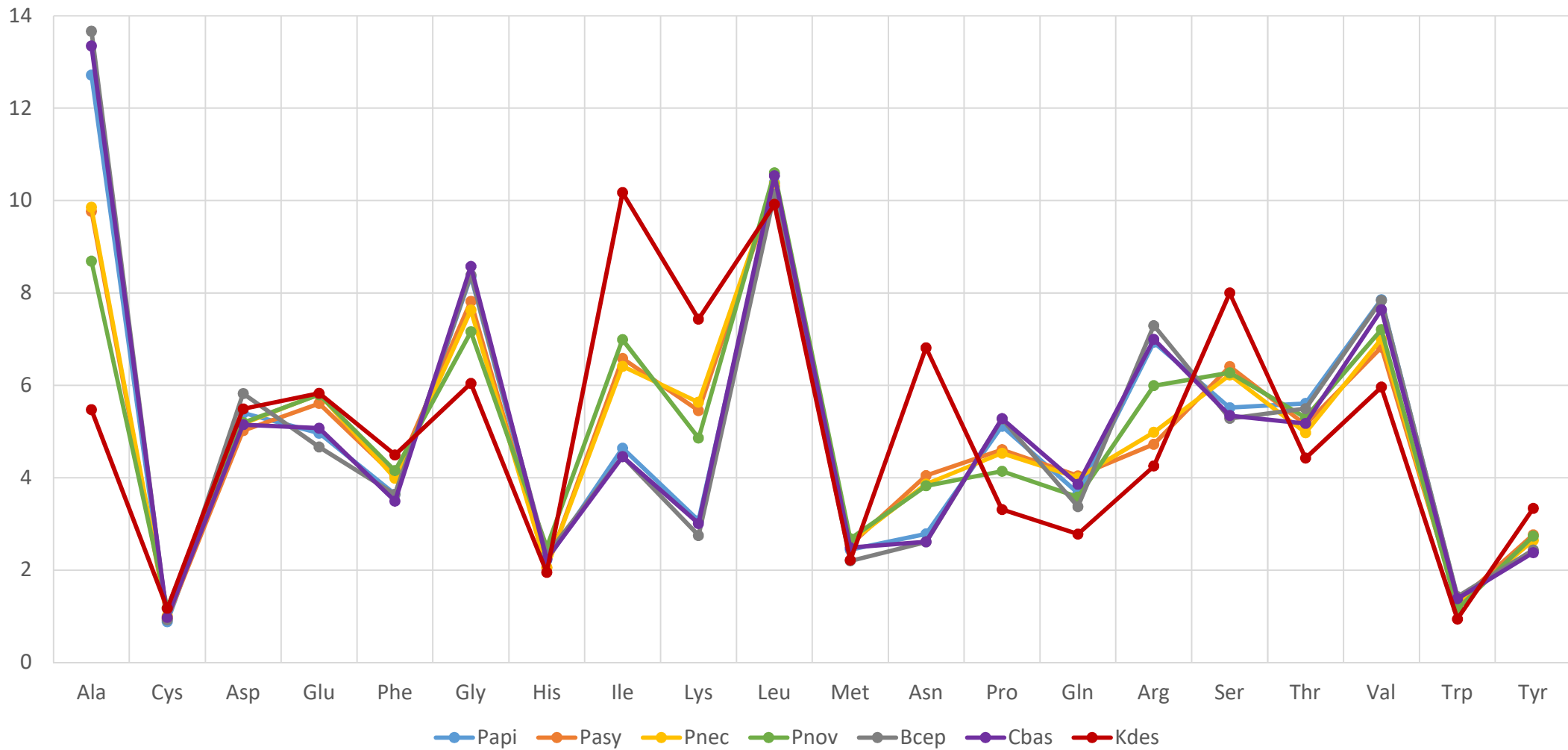

Supplement: FIGURE S4 — Amino acid frequencies. Abbreviations of bacterial species names are as in Supplementary Figure S1. [file Image_4.PDF]

# Relationship of codon usage bias and GC content

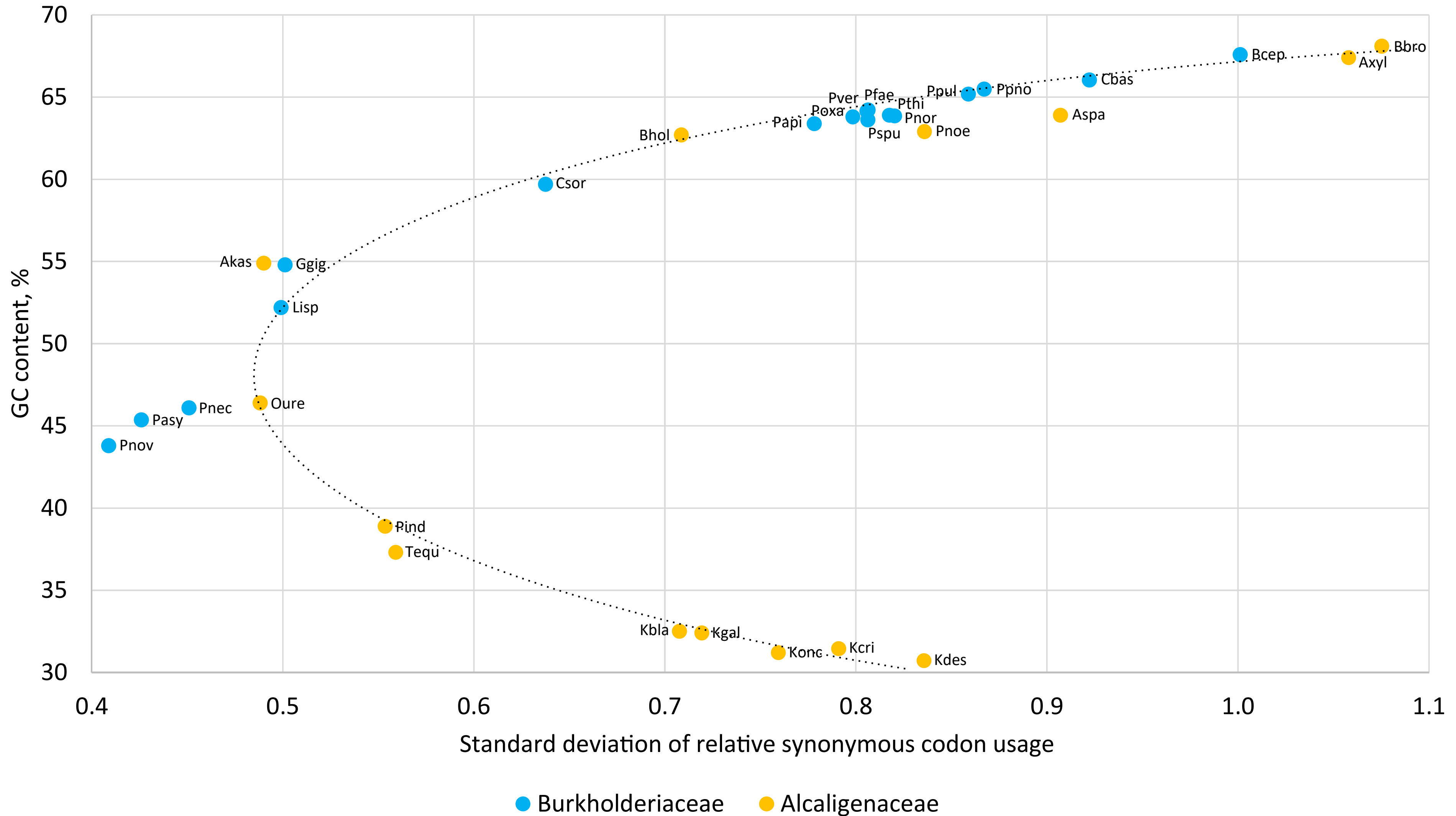

Supplement: FIGURE S5 — Relationship of codon usage bias and GC content in protein coding genes averaged over the whole genome. The dotted line represents a sideways parabola fitting the distribution of values. Akas, Advenella kashmirensis; Tequ, Taylorella equigenitalis; Axyl, Achromobacter xylosoxidans; Aspa, Achromobacter spanius; Bbro, Bordetella bronchiseptica; Bhol, Bordetella holmesii; Pnoe, Pusillimonas noertemannii; Pind, Pelistega indica; Oure, Oligella urethralis; Lisp, Limnobacter sp. CACIAM 66H1; Ggig, Ca. Glomeribacter gigasporarum; Csor, Caballeronia sordidicola. All other abbreviations of bacterial species names are as in Supplementary Figure S1. [file Image_5.PDF]

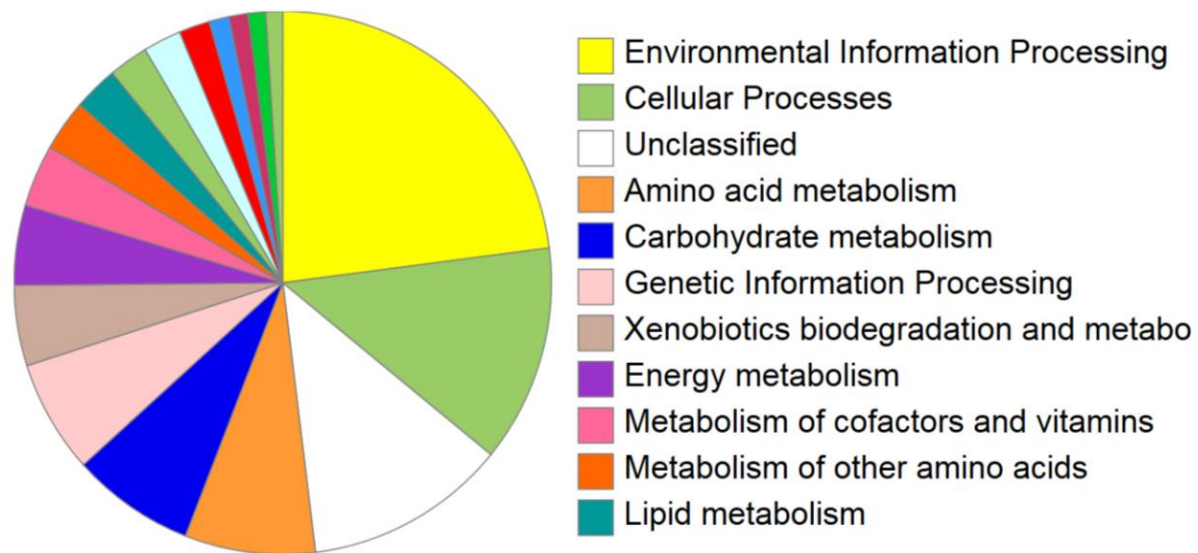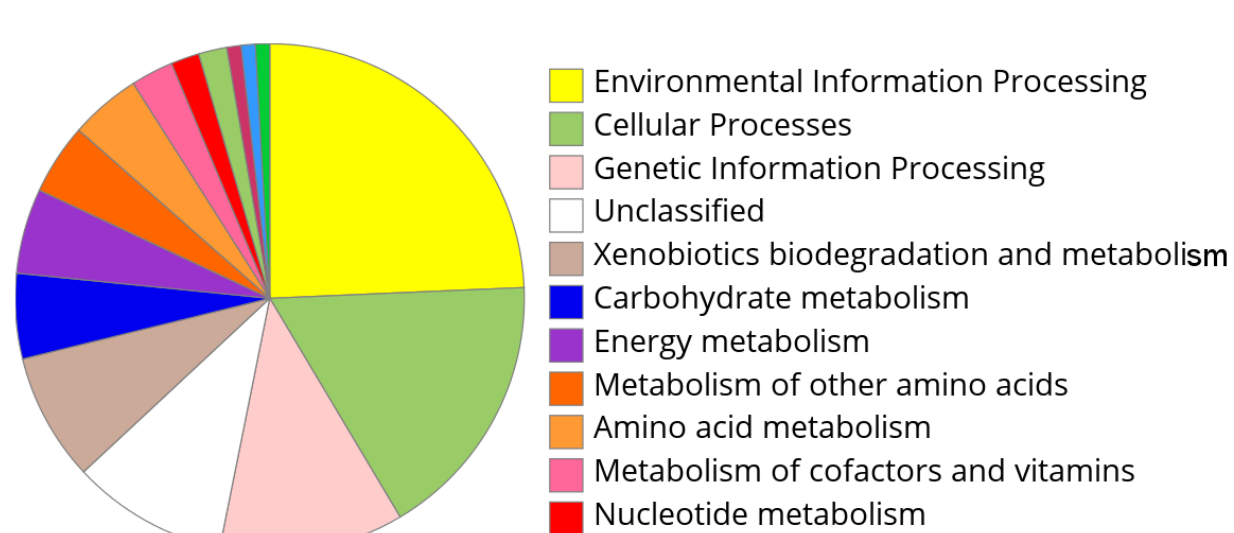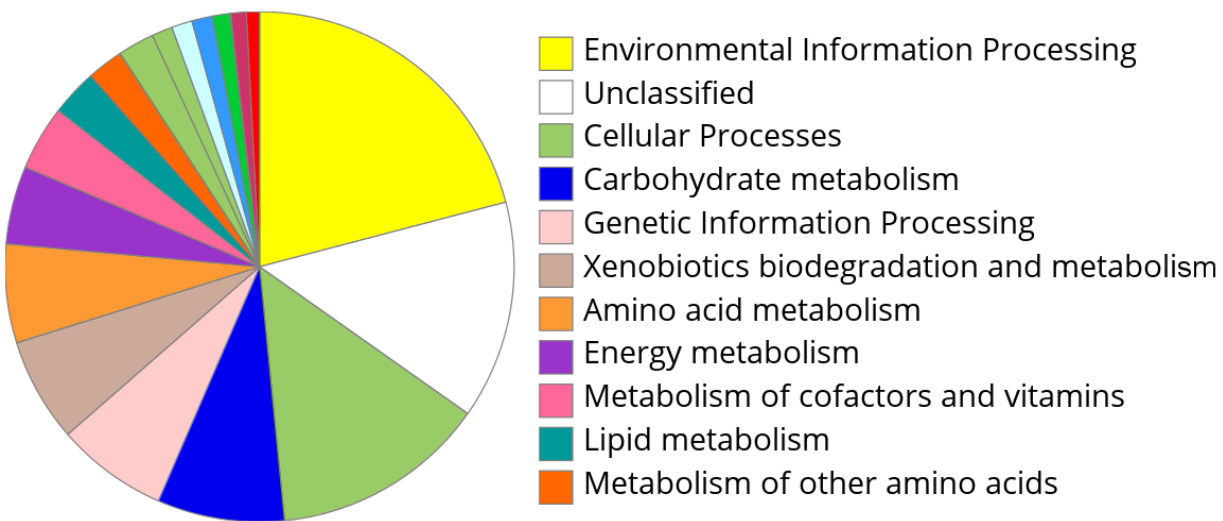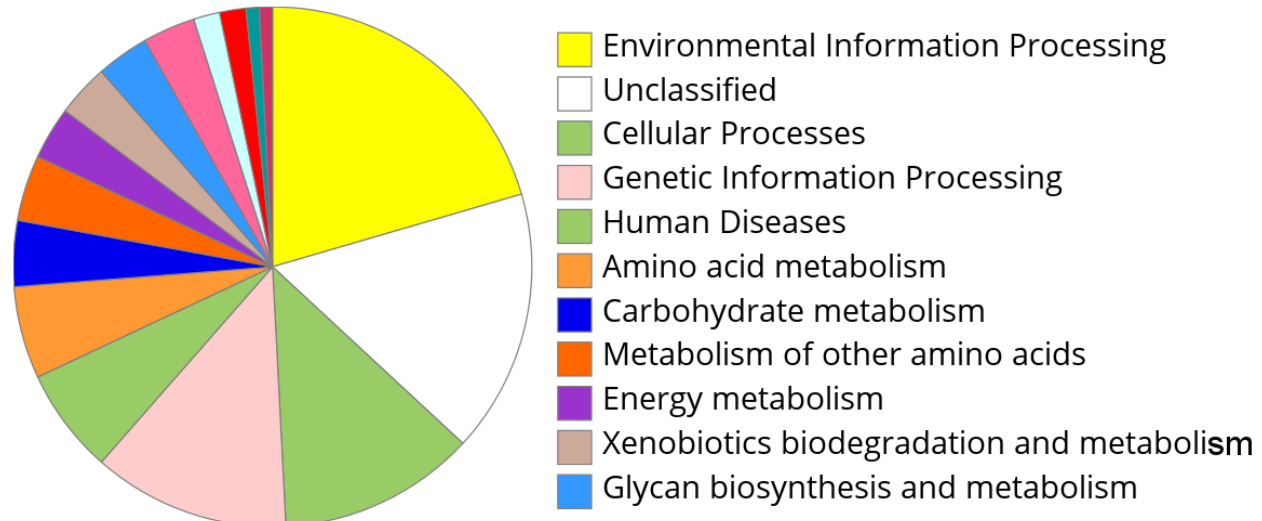

Supplement: FIGURE S7 — A pie chart showing gene annotations for 3,867 OGs lost in Ca. P. novymonadis (Pnov) categorized according to the KEGG Orthology system. Pandoraea pnomenusa (Ppno), Pandoraea norimbergensis (Pnor), and Pandoraea vervacti (Pver) are shown for comparison. [file Image_7.PDF]

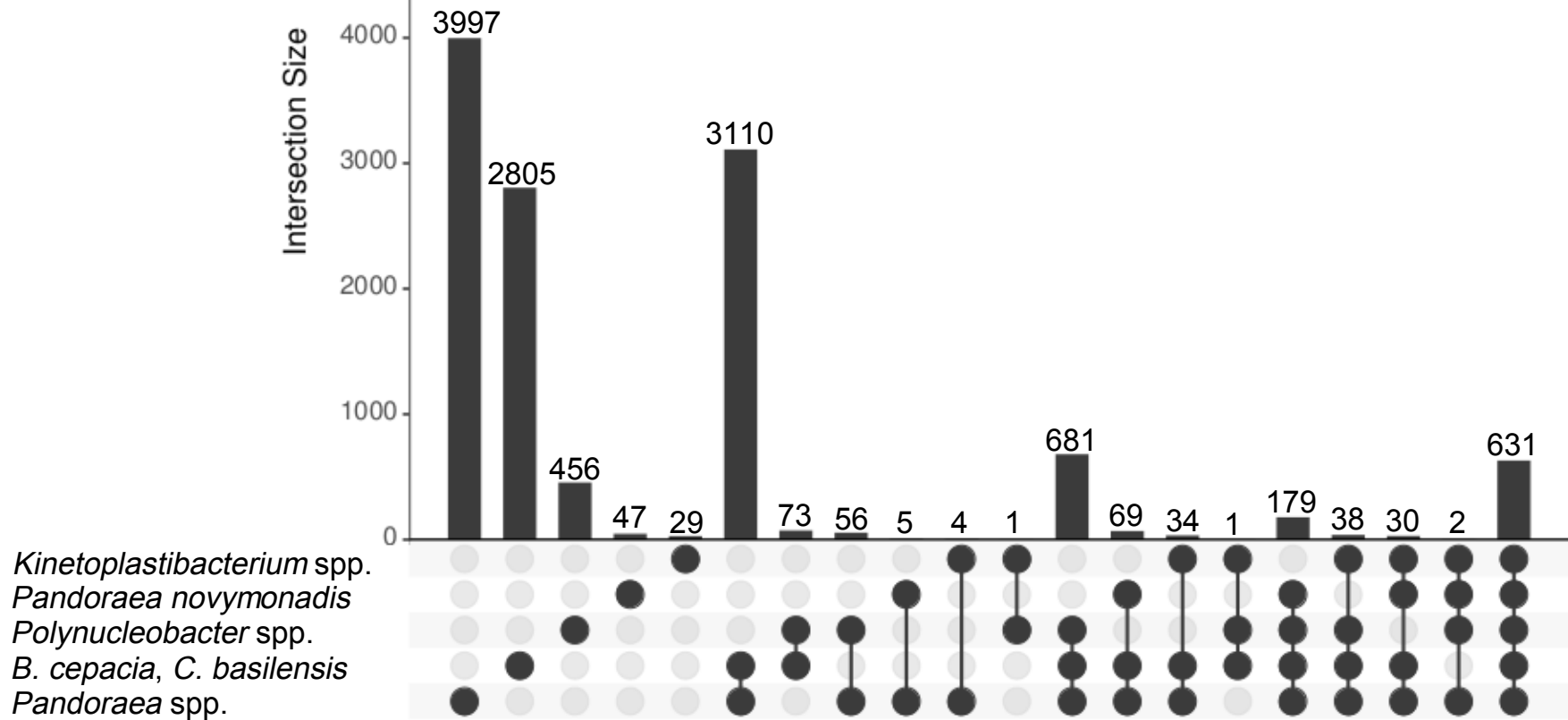

Supplement: FIGURE S8 — Analysis of OGs sharing between free-living Pandoraea spp., Ca. Pandoraea novymonadis, Ca. Kinetoplastibacterium spp., Polynucleobacter spp., Burkholderia cepacia, and Cupriavidus basilensis. OGs were categorized according to their presence in the analyzed species. Depicted bars indicate number of OGs that are unique or shared among the genomes of the organisms listed, as indicated by the black dots. [file Image_8.PDF]

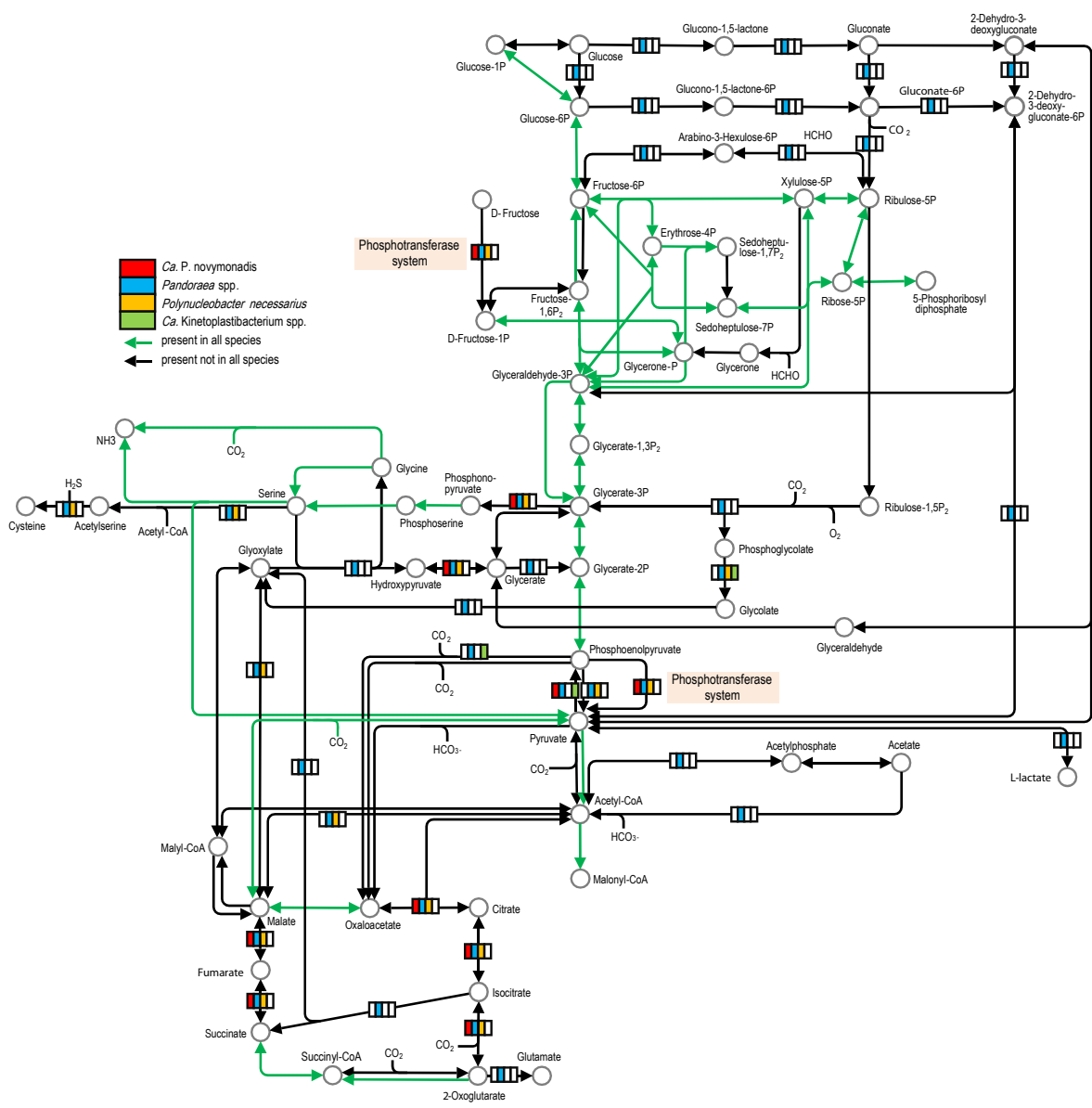

Supplement: FIGURE S9 — Carbon metabolism in Ca. P. novymonadis, free-living Pandoraea spp., Polynucleobacter necessarius, and Ca. Kinetoplastibacterium spp. [file Image_9.PDF]
