# Supplementary material for: Genome of Ca. Pandoraea novymonadis, an Endosymbiotic Bacterium of the Trypanosomatid Novymonas esmeraldas
Source: Front Microbiol. 2017 Oct 4;8:1940. doi: 10.3389/fmicb.2017.01940 (PMC5632650; doi:10.3389/fmicb.2017.01940)
Supplement: FIGURE S6 — Schematic representation of two-way synteny between Ca. P. novymonadis and other Pandoraea spp. with sequenced genomes. The four longest Ca. P. novymonadis contigs are colored according to the legend. Only scaffolds with synteny blocks are shown. Direct synteny blocks are displayed in red, inverted ones – in green. The contigs are drawn proportionately to their actual length. The genomes of Pandoraea spp. shown on the figure are fully assembled to the level of circular chromosomes depicted as the longest colored bars. For some species the shorter colored bars representing plasmids are shown in addition to the chromosomal scaffolds. [file Image_6.PDF]

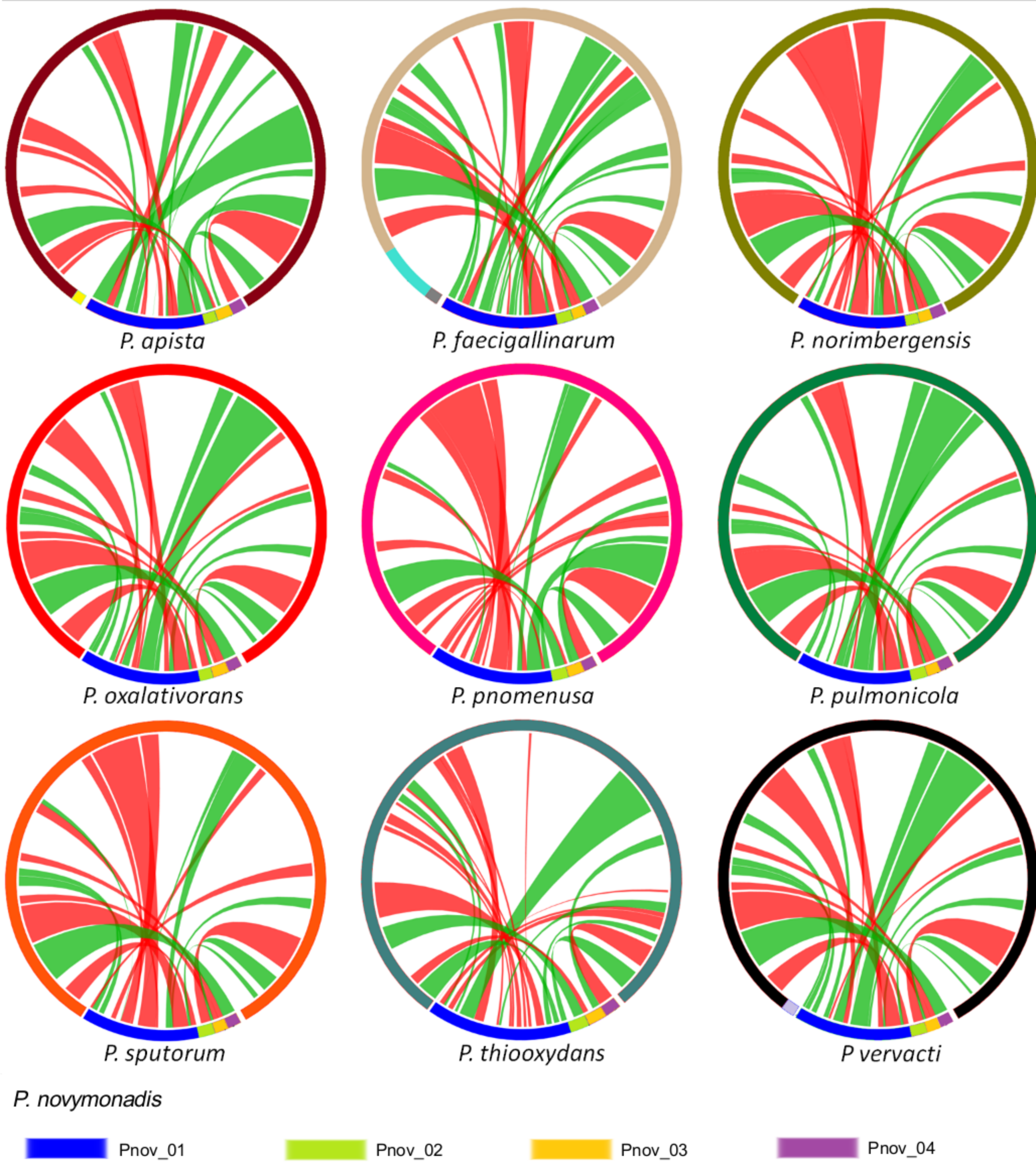
P. apista
P. faecigallinarum
P. norimbergensis
P. oxalativorans
P. pnomenusa
P. pulmonicola
P. sputorum
P. thiooxydans
P vervacti
P. novymonadis
Pnov_01
Pnov_02
Pnov_03
Pnov_04
